# Supplementary material for: Association between Urinary Metabolites and the Exposure of Intensive Care Newborns to Plasticizers of Medical Devices Used for Their Care Management
Source: Metabolites. 2021 Apr 19;11(4):252. doi: 10.3390/metabo11040252 (PMC8073472; doi:10.3390/metabo11040252)

Supplementary file 1. Characteristics of patients enrolled in the ARMED study.

		Center 1	Center 2	Total
**Number of patients enrolled**		53	51	104
**Sexe ratio (M/F)**		35/18	28/23	63/41
**Gestational age**<br>(weeks of amenorrhea ; mean±sd ; median [25th and 75th percentiles])		36.1±4.2<br>37 [33-40]	36.4±4.5<br>38 [34.5-39]	36.2±4.3<br>38 [34-39]
**Birth weight (kg)**<br>(kg ; mean±sd ; median [25th and 75th percentiles])		2.6±0.9<br>2.7 [1.87-3.2]	2.9±0.8<br>2.91 [2.3-3.5]	2.7±0.9<br>2.79 [2.2-3.4]
**Length of study participation**	1 day	53 (100%)	51 (100%)	104 (100%)
	2 days	51 (94%)	34 (67%)	85 (82%)
	3 days	43 (81%)	27 (53%)	70 (67%)
	4 days	33 (62%)	19 (37%)	52 (50%)
	5 days	27 (51%)	8 (16%)	35 (34%)
	6 days	21 (40%)	5 (10%)	26 (25%)

Supplementary file 2. Median urinary levels of Cx-MINP before and after transfusion of patients transfused in center 1. Cx-MINP urinary concentrations (ng/µmol creatinine) of patients from center 1 before and after transfusion sessions (except if a transfusion session was performed on Day 1).

Patient number	*Study day*	*Cx-MeMINP before the transfusion session at the study day*	*Cx-MeMINP after the transfusion session at the study day*
01-04-001	2	0.280	2.823
01-04-001	3	2.823	2.135
01-04-004	2	0.660	0.683
01-04-007	2	0.486	0.685
01-04-023	2	0.440	3.645
01-04-023	3	3.645	21.813
01-04-034	2	0.730	2.260
01-04-035	2	0.405	2.941
01-04-035	3	2.941	18.633
01-04-035	5	23.954	22.580
01-04-037	3	8.169	12.161
01-04-045	2	13.600	17.866
01-04-059	4	17.713	101.886

Supplementary file 3. Extract of the CRF of the patients included in the study.

ARMED

Date : __/__/__

Jour d'étude : J1

Volume d'urines collecté sur 24 heures : mL

Clairance à la créatinine (Cockroft) : mL/min

Nom de l'enquêteur :

Fonction :

☐ Photothérapie : si oui, durée ___h

☐ Incubateur (☐ fermé ☐ ouvert)

Recensement des situations cliniques

☐ DIALYSE ((☐ HD ☐ HF ☐ HDF)

☐ NUTRITION PARENTÉRALE

☐ NUTRITION ENTÉRALE

☐ CEC ☐ ECMO ☐ ECLS

☐ PLASMAPHÉRÈSE

Recensement des DMs utilisés

PERFUSEURS			
Modèle	Fournisseur	Heures de pose	Heures de retrait
☐ Perfusend L86-P *(perfuseur simple)*	SENDAL	__h__	__h__
☐ VLST00 (pompe Volumat)	LÉPINE	__h__	__h__
☐ AX02SF (pompe Alaris)	SENDAL	__h__	__h__
☐ KIS 1X (simple)	DORAN	__h__	__h__
**TRANSFUSEURS**			
☐ Transfusend *(transfuseur simple)*	SENDAL	__h__	__h__
☐ 41403 *(transfuseur avec mesurette)*	SLB MEDICAL	__h__	__h__
**PROLONGATEURS**			
☐ Biocath opaque PO3115 (L150cm)	CAIR	__h__	__h__
☐ Biocath PB3315M *(2,5mm ; L150cm Exsanguinotransfusion)*	CAIR	__h__	__h__
Biocath PB31xxxM<br>☐3101M *(L10cm)* ☐3102M *(L25cm)* ☐3105M ☐3107M *(L50cm)* ☐3110M *(L100cm)* ☐3115M *(L150cm)* ☐3118M *(L180cm)* ☐3120M *(L200cm)*	CAIR	__h__<br>__h__<br>__h__	__h__<br>__h__<br>__h__
☐ Prolongateur avec filtre 0,2µ NEO96E		__h__	__h__
**DISPOSITIFS DE NUTRITION ENTÉRALE**			
☐ Tubulure 777015 (E-pump Kangaroo)	COVIDIEN	__h__	__h__
☐ Tubulure 777007 (E-pump Kangaroo)	COVIDIEN	__h__	__h__
Prolongateur NE Nutrisafe<br>☐368152 (L150cm) ☐368032 (L30cm) ☐53072103 (L30cm)	VYGON	__h__	__h__
☐ Prolongateur bouton GPE ref 8884741821 (bouton Nutriport)	COVIDIEN	__h__	__h__
☐ Prolongateur bouton GPE ref 012412 (bouton Mickey)	ASEPTINMED	__h__	__h__
Canule de prélèvement Nutrisafe<br>☐817002 ☐817202 *(L20cm ; lait)* ☐817302 *(L30cm)* ☐817052 *(L5cm ; médicament)*	VYGON	__h__	__h__
**DISPOSITIFS DE RÉANIMATION**			
CANULES DE TRACHÉOTOMIE			
Shiley<br>- Pédiatriques ss ballonnet: ☐3.0 PED ☐3.5 PED ☐4.0 PED ☐4.5 PED ☐5.0 PED ☐5.5 PED<br>- Pédiatriques avec ballonnet : ☐4.0 PDC ☐4.5 PDC ☐5.0 PDC ☐5.5 PDC	COVIDIEN	__h__	__h__

CRF patient version 1 octobre 2013

Supplementary file 4. Apparent diffusion ability, Dapp (cm/min) of each plasticizer.

	DEHP	DEHT	DINP	DINCH
D_{app}	$4.04.10^{-7}$	$5.69.10^{-8}$	$9.34.10^{-7}$	$2.06.10^{-7}$

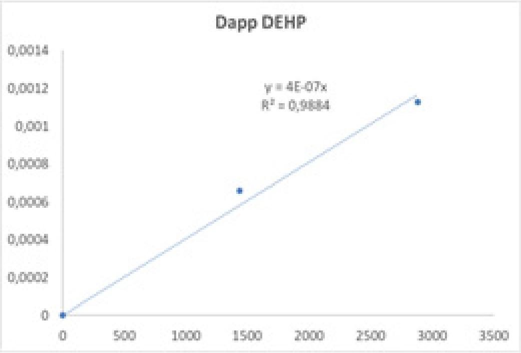

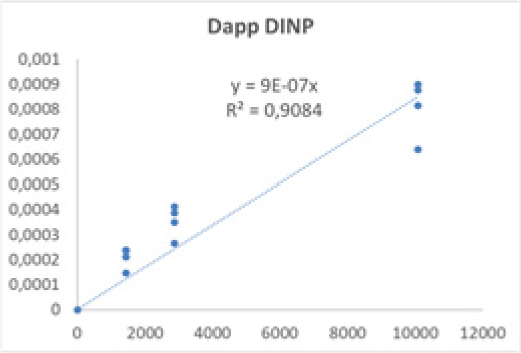

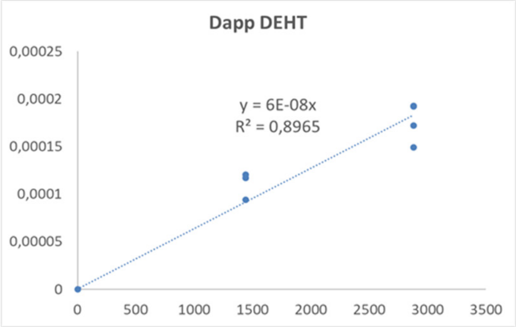

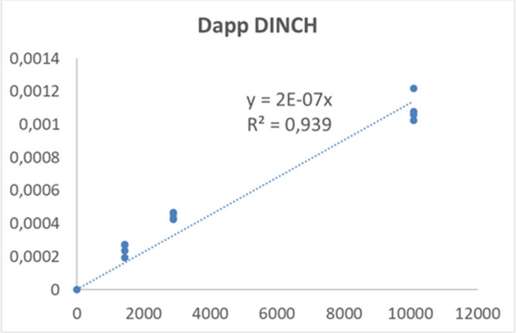

Supplement: Supplementary file 1 [file metabolites-11-00252-s001.zip › metabolites-1173616-supplementary.pdf]
